# The Role of HbA1c as a Positive Perioperative Predictor of Surgical Site and Other Postoperative Infections: An Explorative Analysis in Patients Undergoing Minor to Major Surgery

**DOI:** 10.1007/s00268-021-06368-x

**Published:** 2021-11-08

**Authors:** Andrea Kopp Lugli, Walter R. Marti, Lilian Salm, Edin Mujagic, Marcel Bundi, Marco von Strauss, Evelin Bucheli Laffer, Julia Landin, Christoph A. Fux, Michael Coslovsky, Walter Paul Weber, Christoph Kindler

**Affiliations:** 1 Clinic for Anaesthesia, Intermediate Care, Prehospital Emergency Medicine and Pain Therapy, University Hospital Basel, 4031 Basel, Switzerland; 2 Department of General Surgery, Kantonsspital Aarau AG, Aarau, Switzerland; 3 Clarunis, Department of Visceral Surgery, University Centre for Gastrointestinal and Liver Diseases, St. Clara Hospital and University Hospital Basel, Basel, Switzerland; 4 Department of Anesthesia, Kantonsspital Aarau AG, Aarau, Switzerland; 5 Division of General Surgery, Department of Surgery, University Hospital Basel, Basel, Switzerland; 6 Department of Infectious Diseases and Hospital Hygiene, Kantonsspital Aarau AG, Aarau, Switzerland; 7 Department of Clinical Research, Clinical Trial Unit, University Hospital Basel, Basel, Switzerland

## Abstract

**Background:**

Patients with diabetes mellitus type 2 (DM2) inhere impaired peripheral insulin action leading to higher perioperative morbidity and mortality rates, with hospital-acquired infections being one important complication. This post hoc, observational study aimed to analyze the impact of surgical and metabolic stress as defined by the surrogate marker hemoglobin A1c (HbA1c), in relation to self-reported DM2, on perioperative infection rates in a subcohort of the Surgical Site Infection (SSI) Trial population.

**Methods:**

All patients of the SSI study were screened for HbA1c levels measured perioperatively for elective or emergency surgery and classified according to the American Diabetes Association HbA1c cutoff values. SSI and nosocomial infections, self-reported state of DM2 and type of surgery (minor, major) were assessed.

**Results:**

HbA1c levels were measured in 139 of 5175 patients (2.7%) of the complete SSI study group. Seventy patients (50.4%) self-reported DM2, while 69 (49.6%) self-reported to be non-diabetic. HbA1c levels indicating pre-diabetes were found in 48 patients (34.5%) and diabetic state in 64 patients (46%). Forty-five patients of the group self-reporting no diabetes (65.2%) were previously unaware of their metabolic derangement (35 pre-diabetic and 10 diabetic). Eighteen infections were detected. Most infections (17 of 18 events) were found in patients with HbA1c levels indicating pre-/diabetic state. The odds for an infection was 3.9-fold (95% CI 1.4 to 11.3) higher for patients undergoing major compared to minor interventions. The highest percentage of infections (38.5%) was found in the group of patients with an undiagnosed pre-/diabetic state undergoing major surgery.

**Conclusions:**

These results encourage investment in further studies evaluating a more generous and specific use of HbA1c screening in patients without self-reported diabetes undergoing major surgery.

*Trial registration* Clinicaltrials.gov identifier: NCT 01790529

## Introduction

Patients undergoing surgery are confronted with a surgery-related stress response that induces peripheral insulin resistance. This so-called diabetes of injury correlates with the extent of tissue damage. Additional stress can be inflicted by a diabetic state, which further amplifies the metabolic derangements. The severity of these two stress factors plays a relevant role in outcome, with infection being one of the major complications [1–4].

What are the implications for the treating physician once the patient has been scheduled for surgery, and how may preoperative risk evaluation guide therapy and protect from perioperative harm? The European Society of Anaesthesiologists has defined three severity levels including minor, intermediate, and major surgery to categorize the stress response to surgery [5]. In addition, hemoglobin A1c (HbA1c) as an established indicator of blood glucose control during the previous 3 to 4 months [6] can be easily assessed independent of a patient’s prandial state [7] and serve for diagnosing pre-/diabetes [8, 9]. In diabetic patients undergoing cardiac surgery, HbA1c levels predict intraoperative insulin sensitivity and outcome parameters including infection rate [2]. Suboptimal HbA1c levels (>6% for non-diabetic, >7% for diabetic patients) correlate with higher mortality for both diabetic and non-diabetic patients undergoing major surgery [10–12].

Surgical site infection (SSI) is the most common hospital-acquired infection in surgical patients with relevant impacts on medical costs, hospital stay, and outcome [13, 14]. Patients with diabetes mellitus type 2 (DM2) are more susceptible to infections. Patients with preoperative hyperglycemia without DM2 diagnosis suffer from even higher mortality rates one year after surgery compared to patients with known DM2 [15].

The aim of this post hoc, observational analysis of a subcohort of the SSI Trial [16] was to explore the impact of the levels of (1) surgical stress and (2) metabolic stress as defined by the surrogate marker HbA1c, in relation to self-reported DM2, on perioperative infection rates (including SSI and other infections during hospitalization).

## Methods

### Patients

The previous SSI Trial [16] recruited patients from two tertiary care centers (University Hospital of Basel and Kantonsspital Aarau, Switzerland) undergoing visceral, vascular or trauma surgery in the form of elective or emergency procedures between February 21, 2013 and August 3, 2015. This phase 3 randomized controlled superiority trial in 5175 patients detected no effect of early vs. late timing of surgical antimicrobial prophylaxis on SSI rates. Inclusion and exclusion criteria, as well as the study protocol, were as previously described [16, 17].

The database of the SSI Trial was screened for patients with HbA1c measurements four weeks before or after surgery to be included in this post hoc, observational subcohort analysis. HbA1c levels were not routinely measured as part of the SSI study protocol, but were measured perioperatively only upon specific request of the treating team. HbA1c levels determine diabetic state as proposed by the American Diabetes Association 2017 [18], with levels <5.7% indicating non-diabetic state, 5.7% to 6.5% pre-diabetic state, and ≥6.5% diabetic state. We defined “undiagnosed DM2” as patient self-reporting to be non-diabetic but having HbA1c levels indicative of pre-diabetes or diabetes, “true non-diabetic” and “true diabetic”, respectively, in cases of congruent reported state and metabolic state according to HbA1c level. Patients being previously diagnosed with DM2 but having non-diabetic HbA1c levels were considered to be successfully treated diabetic patients.

All patients with a perioperatively measured HbA1c level were pooled into one study group for this subanalysis regardless of their initial randomly assigned intervention arm for surgical antimicrobial propylaxis timing, as the SSI Trial detected no difference in such timing.

### Definitions

*Infection* The primary end point was the occurrence of any infection (SSI or other nosocomial infection) within 30 days after surgery. The follow-up for detection of any infection was performed by routine clinical rounds and laboratory testing during hospitalization and by telephone assessment 30 days after surgery as previously described [16]. SSI was defined as incisional (superficial/deep), organ or space infection according to the US Centers for Disease Control and Prevention (CDC) criteria published in 1999 [16]. Other nosocomial infections were defined based on the CDC Surveillance Definition of Healthcare-Associated Infection published in 2012 [19] and include urinary tract infection, bloodstream infection, sepsis, skin and soft tissue infection, gastrointestinal infection and pulmonary infection.

*Cardiovascular risk profile* A cardiovascular risk profile was acknowledged in patients having any of the following International Classification of Diseases (ICD) codes registered as secondary diagnosis including I10 (primary arterial hypertension), E78 (hyper-/dyslipidemia), I20-25 (coronary heart disease), and I63-65 (cerebrovascular disease).

*Type of surgery* Type of surgery was assessed according to the preoperative surgical risk estimation stated in the European Society of Cardiology/European Society of Anaesthesiology ESC/ESA guidelines on non-cardiac surgery in three groups: minor, intermediate, and major surgery [5].

### Statistical analysis

The descriptive and exploratory analyses were performed using R version 4.0.4 (RCore Team 2021) [20]. Mean and standard deviations (SD) are provided for continuous variables with a distribution not deviating strongly from normal, and median with the first and third quantile values for those with strong skewness or asymmetry. We report frequency and percentages for nominal and ordinal variables. The low number of events and the small sample size did not allow implementing complex statistical methods. To estimate the difference in odds of infection according to surgical stress level, we fit a logistic regression model implementing the bias reduction method developed by Firth [21] and implemented in the R package ‘brglm’ [22]. This uses a modified-scores approach for estimation, which shrinks estimates and confidence intervals toward the origin compared to maximum likelihood estimates, but provides more stable estimates especially in cases of sparse data or complete separation. This implementation provides a confidence interval based on the union of confidence intervals from the profiling of the ordinary deviance from maximum likelihood fit and by profiling of the penalized deviance for the maximum penalized fit. [22]. We provide the odds ratio (OR) alongside its 95% confidence interval and the *p* value. The *p* value should be seen as a continuous measure of evidence against the null; we do not compare to any predefined thresholds of significance.

## Results

### Patients

HbA1c levels were measured in a total of 139 of 5175 SSI Trial patients [2.7%; 4695 self-reported being non-diabetic, 480 with known DM2 (“Appendix 1”)]. Of the 139 patients with available HbA1c, 69 (49.6%) self-reported being non-diabetic and 70 (50.4%) self-reported DM2. Patient characteristics stratified by self-reported diabetic state are summarized in Table 1. About half of the patients underwent vascular surgery, followed by visceral operations. Patients with self-reported DM2 tended to be older, had higher ASA classifications, and suffered more often from other diseases, as well as fulfilled more often the criteria for a cardiovascular risk profile.

**Table 1:** Patient characteristics stratified by self-reported diabetic state

	Self-reported non-diabetic (*n* = 69; 49.6%)	Self-reported diabetic (*n* = 70; 50.4%)
Age	62.3 (17.3)	66.9 (12.8)
Sex (female/male)	35/34	29/41
BMI (kg m^−2^)	30.7 (8.3)	30.9 (7.6)
HbA1c (%)	5.80 [5.50, 6.20]	7.3 [6.5, 8.1]
ASA classification, *n* (%)		
1	0	0
2	29 (42.0%)	18 (26.1%)
3	39 (56.5%)	49 (71.0%)
4	1 (1.4%)	2 (2.9%)
Surgical division, *n* (%)		
Visceral surgery	24 (34.8%)	17 (24.3%)
Traumatology	7 (10.1%)	15 (21.4%)
Vascular surgery	38 (55.1%)	38 (54.3%)
Surgical modality		
Elective/emergency	60/9	65/5
Type of surgery		
Major surgery (%)	16 (23.2)	23 (32.9)
Minor surgery (%)	53 (76.8)	47 (67.1)
Cardiovascular risk profile (%)	41(59.4)	53 (75.7)
Number of additional diagnoses	3 [1.0, 5.0]	6 [4.0, 8.0]
Smoking (yes/stopped/never/na)	28/15/14/12	26/17/13/14
Duration of surgery (min)	115 [80.0, 167.0]	112 [72.5, 155.0]
Number of infections (%)		
SSI or other nosocomial infections	7 (10.1%)	11 (15.7%)

*HbA1c* Hemoglobin A1c, *ASA* American Society of Anesthesiologists, *BMI* Body mass index, *SSI* Surgical site infection, *na* not available

Values are mean (SD, standard deviation) and [median; IQR, interquartile range]

### Metabolic stress as defined by HbA1c

Patient characteristics stratified according to diabetic state based on HbA1c levels are listed in Table 2. According to HbA1c level, 27 patients (19.4%) were non-diabetic, whereas 48 (34.5%) and 64 patients (46%) were assessed as having a pre-diabetic and diabetic state, respectively. Patients with a pre-diabetic or diabetic state as defined by HbA1c were slightly older and more often male patients. ASA classification and body mass index (BMI) tended to be lowest in non-diabetic and highest in diabetic patients. Furthermore, diabetic patients presented with a higher number of additional diagnoses. Pre- and diabetic patients underwent more vascular surgeries, while non-diabetic patients underwent visceral surgery in almost two thirds of cases.

**Table 2:** Patient characteristics stratified by diabetic state based on HbA1c measurements

	Non-diabetic (*n* = 27; 19.4%)	Pre-diabetic (*n* = 48; 34.5%)	Diabetic (*n* = 64; 46%)
Age	52.3 (19.8)	66.9 (13.7)	68.2 (11.4)
Sex (female/male)	13/14	21/27	30/34
HbA1c (%)	5.40 [5.05, 5.50]	6.00 [5.80, 6.20]	7.40 [6.97, 8.12]
BMI (kg m^−2^)	33.3 (8.1)	28.6 (7.1)	31.3 (8.1)
ASA classification, *n* (%)			
1	0	0	0
2	16 (59.3)	17 (35.4)	14 (22.2)
3	11 (40.7)	30 (62.5)	47 (74.6)
4	0	1 (2.1)	2 (3.2)
Surgical division, *n* (%)			
Visceral surgery	16 (59.3)	10 (20.8)	15 (23.4)
Traumatology	3 (11.1)	7 (14.6)	12 (18.8)
Vascular surgery	8 (29.6)	31 (64.6)	37 (57.8)
Surgical modality			
Elective/emergency	24-Mar	42/6	59/5
Type of surgery			
Major surgery (%)	3 ( 11.1)	12 (25.0)	24 (37.5)
Minor surgery (%)	24 (88.9)	36 (75)	40 (62.5)
Cardiovascular risk profile (%)	14 (59.9)	30 (62.5)	50 (78.1)
Number of additional diagnoses	3 [1, 5.5]	3 [2, 5.2]	6 [4, 8]
Smoking (yes/stopped/never/na)	10/7/5/5	18/9/11/10	26/16/11/11
Duration of surgery (min)	110 [75, 127.5]	112.5 [70, 162.5]	125. [80, 169]
Number of infections (%)			
SSI or other nosocomial infections	1 (3.7%)	8 (16.7%)	9 (14.1%)

*HbA1c* Hemoglobin A1c, *ASA* American Society of Anesthesiologists, *BMI* Body mass index, *SSI* Surgical site infection, *na* not available

Values are mean (SD, standard deviation) and [median; IQR, interquartile range]

A total of 19 patients were operated having an HbA1c > 8% and all of them self-reported a known diabetic state. The HbA1c level in these patients was assessed within a maximum of 14 days with regard to the surgery date. Two surgeries after trauma were emergency procedures. Seventeen interventions involving one trauma, as well as visceral and vascular cases, were planned electively in a short interval as they could not be further postponed from a medical point of view.

### Comparison of knowledge of diabetic state and diabetic state according to HbA1c level

**Fig. 1:**
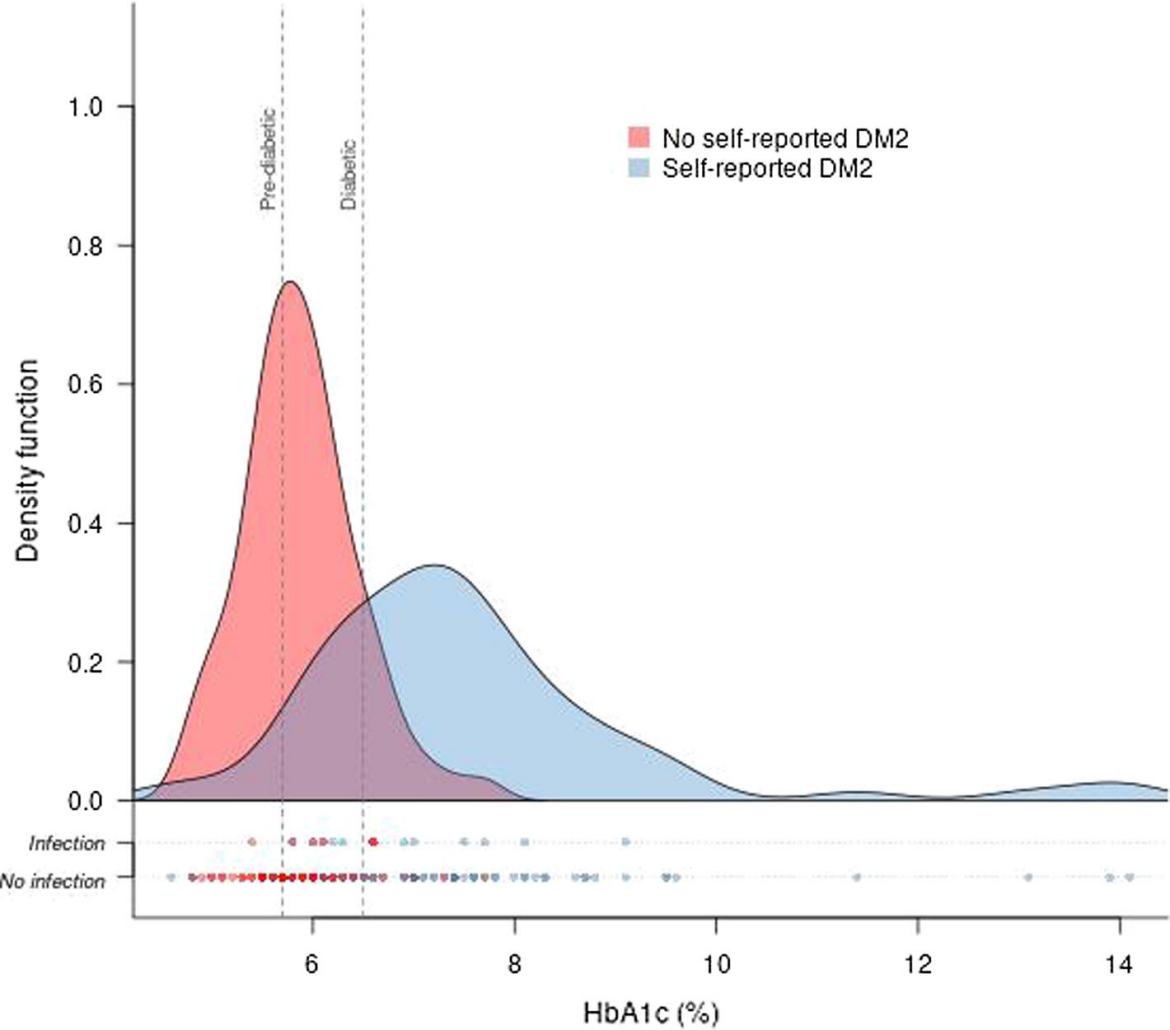
Density distribution of HbA1c levels stratified by self-reported diabetic state (the area under the curve sums to one). Density function of infections (surgical site infection and other nosocomial infections) in relation to HbA1c. Patients with self-reported diabetes mellitus type 2 (blue) and no self-reported diabetes mellitus type 2 (red)

Density distribution of HbA1c levels stratified according to self-reported diabetic state is shown in Fig. 1. Of 69 patients declaring themselves to be non-diabetic, a total of 45 patients had level of HbA1c indicative of pre-diabetes (35 cases) or DM2 (10 cases). The proportion of patients who self-reported no diabetes but were unaware of their metabolic state was 65.2% [95% CI 53.4 to 75.4]. Patients with an undiagnosed pre-/diabetic state were older (68.3 ± 12.8 years) than patients who were true non-diabetic (51.2 ± 19.2 years) and also underwent more vascular surgeries (68.9%). Patients who self-reported their metabolic state correctly as non-diabetic underwent visceral operations in most cases (62.5%). No further relevant differences for patient characteristics were assessed (Table 3). Three patients self-reported being diabetic but had Hba1c levels < 5.7% indicating a successful treatment resulting in 67 true diabetic patients (self-reported state matches HbA1c level).

**Table 3:** Patient characteristics of self-reported non-diabetic patients (*n* = 69) stratified to true non-diabetic patients and patients with an undiagnosed DM2 (self-reported non-diabetic but being pre-diabetic or diabetic according to HbA1c level)

	True non-diabetic (*n* = 24; 34.8%)	Undiagnosed DM2 (HbA1c: pre-diabetic/diabetic) *(n* = 45; 65.2%)
Age	51.2 (19.2)	68.3 (12.8)
Sex (female/male)	12-Dec	23/22
HbA1c (%)	5.40 [5.10, 5.50]	6.10 [5.80, 6.40]
BMI (kg/m^2^)	33.5 (8.1)	29.2 (8.0)
ASA classification, *n* (%)		
1	0 (0)	0 (0)
2	14 (58.3)	15 (33.3)
3	10 (41.7)	29 (64.4)
4	0	1 (2.2)
Surgical division, *n* (%)		
Visceral surgery	15 (62.5)	9 (20.0)
Traumatology	2 (8.3)	5 (11.1)
Vascular surgery	7 (29.2)	31 (68.9)
Surgical modality		
Elective/emergency	21-Mar	39/6
Type of surgery		
Major surgery (%)	3 ( 12.5)	13 (28.9)
Minor surgery (%)	21 (87.5)	32 (71.1)
Cardiovascular risk profile (%)	11 (45.8)	30 (66.7)
Number of additional diagnoses	2.5 [1.0, 5.2]	3.0 [2.0, 5.0]
Smoking (yes/stopped/never/na)	9/6/5/4	19/9/9/8
Duration of surgery (min)	110.0 [75, 129.8]	115 [80, 173]
Number of infections (%)		
SSI or other nosocomial infections	1 (4.2%)	6 (13.3%)

*DM2* diabetes mellitus type 2, *HbA1c* Hemoglobin A1c, *ASA* American Society of Anesthesiologists, *BMI* Body mass index, *SSI* Surgical site infection, *na* not available

Values are mean (standard deviation) or median [interquartile range]

### Level of surgical stress

Due to the low number of patients with a SSI or another nosocomial infection, patients undergoing minor and intermediate surgery were merged into one group (“minor surgery”) and were compared to those undergoing “major surgery.”

A total of 100 so-called minor surgeries were performed in 24 non-diabetic, 36 pre-diabetic and 40 diabetic patients according to their HbA1c level resulting in 21 minor surgeries in true non-diabetic and 13 in undiagnosed DM2 patients. A total of 39 major surgeries were performed in 3 non-diabetic, 12 pre-diabetic and 24 diabetic patients according to their HbA1c level resulting in 3 major surgeries in true non-diabetic and 13 in undiagnosed DM2 patients (Tables 2, 3).

### Association of metabolic and surgical stress with perioperative infection

A total of 18 infections were detected, including 7 in self-reported non-diabetic (10.1% of all self-reported non-diabetic patients) and 11 in self-reported diabetic patients (15.7% of all self-reported diabetic patients) (Table 1). Setting the definition of DM2 according to HbA1c cutoff values, the number of infections was distributed as follows: one infection in the non-diabetic group (3.7%), 8 infections in the pre-diabetic (16.7%), and 9 infections in the diabetic group (14.1%) (Table 2). When using HbA1c levels to define a diabetic state in combination with self-reported DM2 state, one true non-diabetic, 11 true diabetic, and 6 patients with an undiagnosed DM2 suffered from an infection. Therefore, the percentage of infections in patients with undiagnosed DM2 (13.3%) was almost as high as in self-reported diabetic patients (15.7%) and in true diabetic patients, respectively (16.4%) (Tables 1, 3). Almost all patients with an infection had HbA1c levels in the pre-diabetic and diabetic range (Fig. 1).

When using HbA1c levels to assess metabolic state, almost all infectious events occurred in the combined group of pre-/diabetic patients (17 of 112 patients, 15.2%) resulting in an infection risk of about 4.1-fold higher than in non-diabetic patients (1/27, 3.7%) (Table 2 and “Appendix 2”).

Eight of 100 patients (8%) undergoing minor surgery were diagnosed with an infection, while 10 of 39 patients (26%) with major surgery suffered from an infectious event (Table 4). The odds for an infection depending on type of surgery was 3.9-fold higher for major compared to minor surgery (95% CI [1.436, 11.312], *p* = 0.008, *n* = 139).

**Table 4:** Frequency of patients with any nosocomial infection (SSI or other nosocomial infection) according to surgery type and diabetic state based on HbA1c level

Type of surgery	Diabetic state	Total number of patients	No infection (*n* = 121)	Infection (*n* = 18)
Minor	No diabetes	24	24 (100%)	0
	True diabetes	44	37 (84.1%)	7 (15.9%)
	Undiagnosed pre-/diabetes	32	31 (96.9%)	1 (3.1%)
Major	No diabetes	3	2 (66.67%)	1 (33.33%)
	True diabetes	23	19 (82.6%)	4 (17.4%)
	Undiagnosed pre-/diabetes	13	8 (61.54%)	5 (38.46%)

*No diabetes:* true non-diabetic patients and self-reported diabetic patients with a normal HbA1c value indicating successful treatment of diabetes mellitus type 2

*HbA1c* Hemoglobin A1c

Frequency of patients with an infection according to type of surgery and diabetic state as defined by HbA1c level is summarized in Table 4. The percentage of infections in true diabetic patients was comparable for minor (15.9%) and major (17.4%) surgery. The proportion of infections in patients unaware of their diabetic state undergoing major surgery was higher (38.5%) compared to such patients undergoing minor surgery (3.1%). None of the three patients self-reporting a diabetic state and having an HbA1c level indicating successful DM2 treatment suffered from an infection.

## Discussion

This post hoc, explorative observational study of a SSI Trial subcohort assessed the combined effects of surgical and metabolic stress as defined by HbA1c level on risk for in-hospital infection. As expected, self-reported diabetic patients suffered from higher infection rates independent of the type of surgery. The impact of major surgical stress on infection risk was 3.9-fold higher compared to minor surgery. In patients with undiagnosed pre-/diabetic state undergoing major operations, the impact of surgical stress increased the event rate of infection to 38.5% compared to 3.1% for minor surgery.

DM2 has been shown to negatively affect perioperative outcome, with higher rates of infection being a reason for major complications [1]. Therefore, preoperative risk assessment and evaluation of prognostic metabolic biomarkers are of utmost importance. Although HbA1c levels are easy to measure indicators of blood glucose control, they may not reflect actual insulin resistance or harmful blood glucose variability. Nevertheless, they correlate with perioperative mortality and morbidity including infections [12, 23, 24].

Several large cohort studies have investigated the effects of perioperative insulin resistance on outcome in various surgical populations [2, 12]. Despite the relatively small numbers of patients, our study offers quite an unique opportunity to explore interactions of self-reported and newly diagnosed DM2 (as defined by HbA1c level) with the stress response to different degrees of surgical impact and the risk of infection. A relevant percentage of patients (65.2%) self-reporting a non-diabetic state were newly diagnosed as pre-/diabetic with respect to their HbA1c levels. The HbA1c-test population compared to the complete SSI trial involved patients of older age, higher ASA class, higher BMI, with a longer smoking history, and a higher percentage of elective surgeries, suggesting these parameters may have guided preoperative assessment strategies.

As expected, patients with self-reported DM2 showed a higher risk for perioperative infection compared to non-diabetic patients in our study population. Furthermore, when using HbA1c levels to assess metabolic state, almost all infectious events (17 of 18) occurred in the combined group of pre-/diabetic patients resulting in an infection risk of about 4.1-fold higher than in non-diabetic patients. When stratifying patients into true DM2 and patients with undiagnosed DM2 according to their HbA1c levels, the two most notable findings appeared in patients unaware of their metabolic derangement. Their percentage of infections (13.3%) almost reached both the level of infections of self-reported DM2 patients (15.7%) and of true diabetic patients (16.4%). In addition and of special interest, their odds of infection were mostly driven by the type of surgery. A potential reason for these findings could be that patients undergoing surgery suffer from stress-induced hyperglycemia and catabolism. These metabolic derangements are pronounced in DM2 patients leading to higher rates of perioperative complications including infections, particularly SSIs, and increased in-hospital mortality [1]. Furthermore, the stress response to surgery parallels the extent of tissue injury, leading to pronounced hyperglycemia and catabolism, both of which impact postoperative morbidity and mortality [25]. Several studies have focused on the relative impact of stress-induced hyperglycemia on perioperative outcome [15, 26]. In a retrospective analysis [15] including a cohort of more than 61,000 patients undergoing elective non-cardiac surgery, Abdelmalak et al. reported key points which are in line with our results. Our relative high number of patients with pre-/diabetic state suffering from postoperative infections is in accordance with the conclusion of these authors that the risk of infection and possibly other adverse outcomes can only be reduced if perioperative hyperglycemia is treated. Furthermore, surgical patients with preoperative hyperglycemia, but without the diagnosis of diabetes, had a higher mortality one year after surgery in Abdelmalak’s study. Therefore, these authors also propose routine preoperative measurement of HbA1c in suspected diabetics in order to improve care.

There are several limitations to our analyses. The number of patients in whom HbA1c level was measured resulted in a too small number to allow full investigation of various aspects such as controlling for confounding with other variables or examining interactive effects and performing further subgroup analyses, which introduced a selection bias in our analysis. Furthermore, HbA1c levels were not measured in a standardized manner in every patient in the SSI Trial due to clinical feasibility and costs. The included HbA1c measurements occurred pre-, as well as postoperatively, during a time period of four weeks with respect to the day of surgery. However, as HbA1c reflects metabolic state of the last two to four months, we deemed these measurements to be acceptable for this hypothesis-generating analysis. In addition, three patients received red blood cell transfusions intraoperatively, but this introduces no bias as each had their HbA1c levels measured before the day of surgery.

Despite these limitations, the high number of newly diagnosed pre-/diabetic patients proposes the clinically driven screening to have correctly focused on potential patients at risk. Clinicians are inherently more inclined to monitor and treat patients with known DM2 than those reporting to be non-diabetic. Patients who are pre-/diabetic according to HbA1c levels and have no apparent clinical signs of DM2 are particularly at risk of receiving irregular blood glucose monitoring and, therefore, inadequate consecutive treatment. It might be further argued that the detrimental effect of pronounced glucose variability [27] in unknown DM2 patients may pose an additional risk of infection. According to our data, patients undergoing major surgery might benefit most from a generous screening for DM2, as stress-induced hyperglycemia parallels the extent of tissue injury during surgery, adding further metabolic impact to an already pre-/diabetic state.

In summary, easily accessible preoperative screening tools for diabetic metabolism offer one opportunity to identify patients at risk and to better control perioperative glucose homeostasis and finally outcome [15]. The presented results encourage not only investment in further studies in this field but also heightened attention for further stratifying perioperative care. Firstly, patients with diagnosed DM2 require good perioperative blood glucose measurement and treatment to counteract the increased risk of infection, and secondly, patients reporting a non-diabetic state deserve a more generous HbA1c screening especially when undergoing major surgery or presenting with a cardiovascular risk profile.

## Appendix 1

Demographics of the complete SSI trial population stratified by self-reported diabetic state.

**Table:**

	Self-reported non-diabetic (*n* = 4695; 90.7%)	Self-reported diabetic (*n* = 480, 9.3%)
Age	56.1 (18.8)	67.3 (12.6)
Sex (female/male)	2185/2510	188/292
BMI (kg m^−2^)	26.7 (6.8)	30.7 (6.9)
ASA classification, *n* (%)		
1	931 (19.8%)	1 (0.2%)
2	2556 (54.5%)	176 (36.7%)
3	1161 (24.7%)	287 (59.9%)
4	46 (1.0%)	15 (3.1%)
Surgical division, *n* (%)		
Visceral surgery	2285 (48.7%)	226 (47.1%)
Traumatology	1875 (39.9%)	130 (27.1%)
Vascular surgery	535 (11.4%)	124 (25.8%)
Surgical modality		
Elective/emergency	3847/848	408/72
Cardiovascular risk profile	1328	335
Number of additional diagnoses	2.9 (2.4)	5.2 (2.2)
Smoking (yes/stopped/never/na)	1167/631/1017/1867	111/121/86/160
Duration of surgery (min)	87 [59.0, 135.0]	100 [65.8, 152.2]

*ASA* American Society of Anesthesiologists, *BMI* Body mass index, *SSI* Surgical site infection, *na* Not available

Values are mean (SD, standard deviation) and [median; IQR, interquartile range]

## Appendix 2

Distribution of infections according to diabetic state based on HbA1c levels.

**Table:**

Type of infection	Non-diabetic *n* = 27	Pre-diabetic *n* = 48	Diabetic *n* = 64
None	26	40	55
Bloodstream infection, sepsis	0	2	0
Urinary tract infection	0	3	4
Gastrointestinal infection	1	1	2
Pulmonary infection	0	1	0
Surgical site infection			
	0	1	3

*HbA1c* Hemoglobin A1c
